# D-Cateslytin, a new antimicrobial peptide with therapeutic potential

**DOI:** 10.1038/s41598-017-15436-z

**Published:** 2017-11-09

**Authors:** Abdurraouf Zaet, Pauline Dartevelle, Fadoua Daouad, Claire Ehlinger, Fabienne Quilès, Grégory Francius, Christian Boehler, Camille Bergthold, Benoît Frisch, Gilles Prévost, Philippe Lavalle, Francis Schneider, Youssef Haïkel, Marie-Hélène Metz-Boutigue, Céline Marban

**Affiliations:** 1 Université de Strasbourg, Faculté de Chirurgie Dentaire, 3 rue Sainte Elisabeth, 67000 Strasbourg, France; 2 Inserm UMR 1121, Fédération de Médecine Translationnelle de Strasbourg, 11 rue Humann, 67085 Strasbourg, France; 3 Université de Lorraine, Laboratoire de Chimie Physique et Microbiologie pour l’Environnement, LCPME, UMR 7564, 54600 Villers-lès, Nancy, F-54600 France; 4 CNRS, Laboratoire de Chimie Physique et Microbiologie pour l’Environnement, LCPME, UMR 7564, 54600 Villers-lès, Nancy, F-54600 France; 5 Laboratoire de Conception et Applications des Molécules Bioactives, Faculté de Pharmacie, UMR 7199 CNRS/Université de Strasbourg, 74 Route du Rhin, 67401 Illkirch, France; 6 Université de Strasbourg, CHRU Strasbourg, Fédération de Médecine Translationnelle de Strasbourg, VBP EA/7290, 67000 Strasbourg, France; 7 Service de Réanimation Médicale, Hôpital de Hautepierre, Hôpitaux Universitaires de Strasbourg, Strasbourg, France

## Abstract

The rise of antimicrobial resistant microorganisms constitutes an increasingly serious threat to global public health. As a consequence, the efficacy of conventional antimicrobials is rapidly declining, threatening the ability of healthcare professionals to cure common infections. Over the last two decades host defense peptides have been identified as an attractive source of new antimicrobials. In the present study, we characterized the antibacterial and mechanistic properties of D-Cateslytin (D-Ctl), a new epipeptide derived from L-Cateslytin, where all L-amino acids were replaced by D-amino acids. We demonstrated that D-Ctl emerges as a potent, safe and robust peptide antimicrobial with undetectable susceptibility to resistance. Using Escherichia coli as a model, we reveal that D-Ctl targets the bacterial cell wall leading to the permeabilization of the membrane and the death of the bacteria. Overall, D-Ctl offers many assets that make it an attractive candidate for the biopharmaceutical development of new antimicrobials either as a single therapy or as a combination therapy as D-Ctl also has the remarkable property to potentiate several antimicrobials of reference such as cefotaxime, amoxicillin and methicillin.

## Introduction

The discovery of antimicrobials to treat infectious diseases is one of the greatest achievements of modern medicine. However, excessive and inappropriate use of antimicrobials fosters the emergence and spread of antimicrobial-resistant microorganisms. Indeed, infections caused by antimicrobial-resistant microorganisms also known as “superbugs” often no longer respond to conventional treatments, thereby extending the duration of the disease related to infection and even lead to patient death^1,2^. Antimicrobial-resistant microorganisms, including multidrug-resistant types, are often responsible for healthcare-associated infections and constitute a serious threat to public health worldwide, specifically among vulnerable populations such as critically ill patients^3^. Infections caused by Gram-negative bacteria are a particular concern for public health because these microorganisms are so versatile that they can exchange genetic material and rapidly deploy an arsenal of resistance mechanisms, particularly under selective pressure^4^. Especially, this phenomenon resulted in a drastic increase in the prevalence of *Escherichia coli* multidrug-resistant (*E*. *coli* MDR) strains and the onset of healthcare-associated urinary tract or bloodstream infections^5–8^.

Novel classes of antimicrobials were rare in the past thirty years and of sharp administration. Specifically, the discovery of fluoroquinolones in the 1970s brought to an end the portfolio of antimicrobials against Gram-negative bacteria^9^. Nevertheless, antimicrobial therapy remains the prophylactic and curative practice most commonly used to fight against infections in the city and the hospital. However, due to the emergence of selected antimicrobial-resistant microorganisms and the lack of new antimicrobials on the market, we are now facing the possibility of a future without effective antimicrobials for treating bacterial infections. As a consequence, there is a persisting and urgent medical need to develop new antibacterial compounds.

Over the last two decades, host defence peptides (HDPs) have emerged as new attractive candidates in the development of novel anti-bacterial treatments, specifically for antimicrobial-resistant infections^10^. The benefits of using HDPs are that they act by disrupting the bacterial membranes, a mechanism that is fast and non-specific. Therefore bacteria are not prone to develop high-level resistance towards these compounds in the same extent as towards conventional antimicrobials^11^. Moreover they display a broad-spectrum of pathogens, including multidrug resistant Gram-positive and negative bacteria^12^. HDPs are usually rather short (12–50 amino acids), cationic and amphiphilic with a broad diversity in their secondary structure and well preserved during evolution. HDPs are naturally present in tissues frequently exposed to pathogens, such as the skin, lungs, and gastrointestinal tract. Besides their broad spectrum of antimicrobial properties, they also exhibit significant immunomodulatory effects^13^.

Among all isolated and characterized HDPs, Cateslytin (Ctl) constitutes an excellent candidate for the development of a new class of antimicrobials. Indeed, Ctl is short and linear (15 amino acids) and therefore very easy to synthesize for a minimal cost. Moreover, it is stable at high temperature and low pH. Ctl results from the proteolysis of chromogranin A, an acidic protein stored in the secretory vesicles of numerous neuroendocrine and immune cells and is released upon stress in most of the body fluids^14–17^. In addition to its antibacterial properties, Ctl is also a potent antifungal agent^18,19^.

In the present study, we report the biological characterization of D-Ctl, a new epipeptide derived from L-Ctl, where all L-amino acids were replaced by D-amino acids (patent application: EP16 306539.4). Using various approaches including microbiology (broth microdilution assays), cell biology (viability and cytokine release assays) and microscopy (atomic force microscopy, epiflorescence microscopy, ATR-FTIR spectroscopy), we characterized the biological and mechanical properties of D-Ctl compared to its conformer L-Ctl. Overall, D-Ctl emerges as a potent, safe and stable antimicrobial that damages bacterial cell walls and still not suffer of any microbial resistance.

## Results

### D-Ctl is an efficient antimicrobial agent against various bacterial strains

One of the downfalls of the use of therapeutic peptides relies on their lack of proteolytic stability towards proteases. One way of controlling the stability of a therapeutic peptide is to synthesize its epimer, which has the same sequence as the parent peptide with all levogyre (L) amino acids replaced by dextrogyre (D) amino acids. Such peptides are more resistant to proteolysis, hence increasing their half-lives and bioavailability. Therefore, we synthesized D-Ctl and compared its respective antibacterial efficiency with L-Ctl. To this aim, we used a panel of Gram-negative strains: *Escherichia coli* wild type, *Escherichia coli* multidrug resistant (*E*. *coli* MDR), *Prevotella intermedia*, *Fusobacterium nucleatum* and Gram-positive strains: *Staphylococcus aureus* Methicillin Sensitive (MSSA), *Staphylococcus aureus* Methicillin Resistant (MRSA), *Parvimonas micra*. This panel includes facultative and strict anaerobes **(**Table 1 **)**. The antibacterial activity of D-Ctl versus L-Ctl was assessed by the measurement of their MIC (Minimal Inhibitory Concentration) defined as the lowest concentration of peptide able to inhibit 100% of the inoculum. Depending on the bacterial species, the MIC of D-Ctl ranged between 8 and 24 μg/mL **(**Table 1 and Supplementary Figure S1 **)**. D-Ctl was specifically efficient against *P*. *intermedia* with a MIC of 10 μg/mL and *E*. *coli* with a MIC of 8.0 μg/mL for *E*. *coli* wild type and 8.4 μg/mL for *E*. *coli* MDR. Overall, the MIC of D-Ctl was 2 to 18 times lower than the one of L-Ctl **(**Table 1 and Supplementary Figure S1 **)**.

**Table 1:** Antibacterial activity of D-Ctl compared to L-Ctl.

Pathogen	Gram	Respiratory type	MIC (peptide)		Antibiotic of reference	
			L-Ctl (µg/mL)	D-Ctl (µg/mL)	Name	(µg/mL)
*Escherichia coli* (ATCC 25922)	−	Facultative anaerobe	75	8.0	Ampicillin	7.0
					Kanamycin	21
*Escherichia coli* (MDR) (K-12)	−	Facultative anaerobe	150	8.4	Cefotaxime	0.1
*Fusobacterium nucleatum* (ATCC 49256)	−	Obligate anaerobe	125	22	Amoxicillin	0.6
*Prevotella intermedia* (ATCC 49046)	−	Obligate anaerobe	149	10	Amoxicillin	0.5
*Parvimonas micra* (ATCC 33270)	+	Obligate anaerobe	120	23	Amoxicillin	0.5
*Staphylococcus aureus* (MSSA) (ATCC 25923)	+	Facultative anaerobe	40*	24	Methicillin	1.2
*Staphylococcus aureus* (MRSA) (S1)	+	Facultative anaerobe	37*	18	Vancomycin	0.8

The percentage of growth inhibition of the indicated pathogens in the presence of different concentrations of D-Ctl or L-Ctl was determined by broth microdilution assays. Each MIC, defined as the lowest concentration of a drug able to inhibit 100% of a bacterial inoculum, was determined using a modified Gompertz function. Experiments were performed with biological replicates. *Values obtained from Aslam *et al*.^18^.

We then compared the MIC of D-Ctl with the MIC of antimicrobials of reference. Interestingly, the antimicrobial activity of D-Ctl on *E*. *coli* was comparable to that of ampicillin and kanamycin and could therefore constitute an alternative treatment for *E*. *coli* infections **(**Table 1 and Supplementary Figure S2 **)**. Regarding the others species tested, the antimicrobials of reference were still more efficient than D-Ctl **(**Table 1 and Supplementary Figure S2 **)**.

### D-Ctl is a potentiator for numerous antimicrobials of reference

We then investigated whether D-Ctl could potentiate the antibacterial effect of several antimicrobials of reference, specifically methicillin and vancomycin extensively prescribed to treat *S*. *aureus* infections, amoxicillin recommended in numerous infections including periodontal infections and cefotaxime often used as second intention treatment against *E*. *coli* resistant strains. According to the European Committee on Antimicrobial Susceptibility Testing^20^, the effect of a combination between two antibacterial compounds can be evaluated by their FICI (Fractional Inhibitory Concentration Index). The FICI consists of the sum of the FICs of both antibacterial agents: FIC index = FIC_antimicrobial_ + FIC_D-Ctl_. For each compound, the FIC was determined as the ratio between the MIC of the compound in combination (MIC_combination_) and the MIC of the compound acting alone (MIC_alone_). On the basis of their FIC index, each combination was categorized as synergistic (≤0.5), additive (>0.5 to 1), indifferent (>1 to <4) or antagonistic (≥4).

For each strain, the MICs of D-Ctl and the antimicrobial of reference were evaluated (MIC_alone_) **(**Table 2 and Supplementary Figures S1 and S2 **)**. Then, different combinations of D-Ctl and the antimicrobial of reference were tested in order to determine the MIC_combination_. The FICI was then calculated as described above. We observed a synergistic effect between D-Ctl and amoxicillin for *P*. *micra* and *P*. *intermedia* and an additive effect for D-Ctl and cefotaxime, methicillin and amoxicillin on *E*. *coli* MDR, MSSA and *F*. *nucleatum*, respectively **(**Table 2 and Supplementary Figure S3 **)**. Regarding MRSA, no potentiator effect was highlighted between D-Ctl and methicillin **(**Table 2 and Supplementary Figure S3 **)**. Altogether, D-Ctl also emerges as an effective potentiator for several antimicrobials currently prescribed in clinic to fight severe bacterial infections.

**Table 2:** Antibacterial activity of D-Ctl in combination with conventional antimicrobials.

Pathogens	Combination	MIC _alone_ (µg/mL)	MIC _combination_ (µg/mL)	FIC	FICI	Effect
*Escherichia coli* MDR	D-Ctl	8.4	4.2	0.5	1.0	Additive
	Cefotaxime	0.1	0.05	0.5		
*Fusobacterium nucleatum*	D-Ctl	22	11	0.5	1.0	Additive
	Amoxicillin	0.6	0.3	0.5		
*Prevotella intermedia*	D-Ctl	10	2.5	0.25	0.5	Synergistic
	Amoxicillin	0.5	0.125	0.25		
*Parvimonas micra*	D-Ctl	23	5.8	0.25	0.5	Synergistic
	Amoxicillin	0.5	0.125	0.25		
*Staphylococcus aureus* (MSSA)	D-Ctl	24	12	0.5	0.75	Additive
	Methicillin	1.2	0.3	0.25		
*Staphylococcus aureus* (MRSA)	D-Ctl	18	18	1	2	Indifferent
	Vancomycin	0.8	0.8	1		

The percentage of growth inhibition of the indicated pathogens in the presence of different concentrations of antimicrobials was determined by broth microdilution assays. The MICs of each drug were used to calculate the FIC index of each combination. Each experiment was performed at least in duplicate.

### Unlike ampicillin and cefotaxime, D-Ctl does not trigger resistance in *E*. *coli*

**Figure 1:**
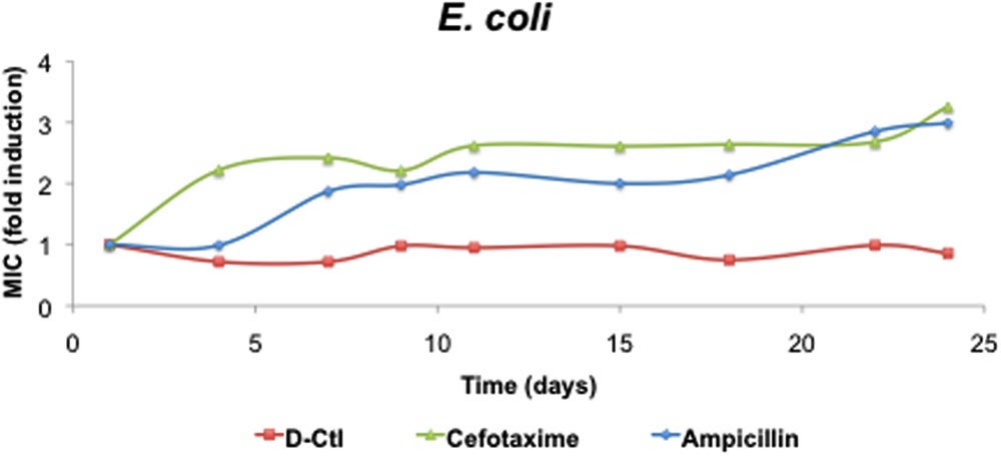
Resistance acquisition assay of *E*. *coli* in the presence of D-Ctl compared to ampicillin and cefotaxime. The *E*. *coli* wild-type strain was cultured in the presence of ½ MIC of the antibacterial agent for 24 days. The fold change in MIC was evaluated at the indicated days.

To assess whether *E*. *coli* would develop resistance under a selective pressure, we cultured *E*. *coli* wild type in the presence of sub-MIC concentrations of D-Ctl (½ MIC), ampicillin or cefotaxime for 24 days. Interestingly, *E*. *coli* failed to generate mutants of resistance as its MIC remained stable for the whole duration of the culture **(**Fig. 1 **)**. In contrast, the MICs of ampicillin and cefotaxime, two antimicrobials of reference used to treat *E*. *coli* infections, rapidly increase over the course of the culture to reach 3x MIC at day 24 **(**Fig. 1 **)**.

### D-Ctl is not cytotoxic and does not elicit cytokine release

In order to investigate whether D-Ctl would be a good lead compound for the development of a new antimicrobial, we assessed several safety issues such as its haemolytic activity, cytotoxicity and immunogenicity through cytokine release.

**Figure 2:**
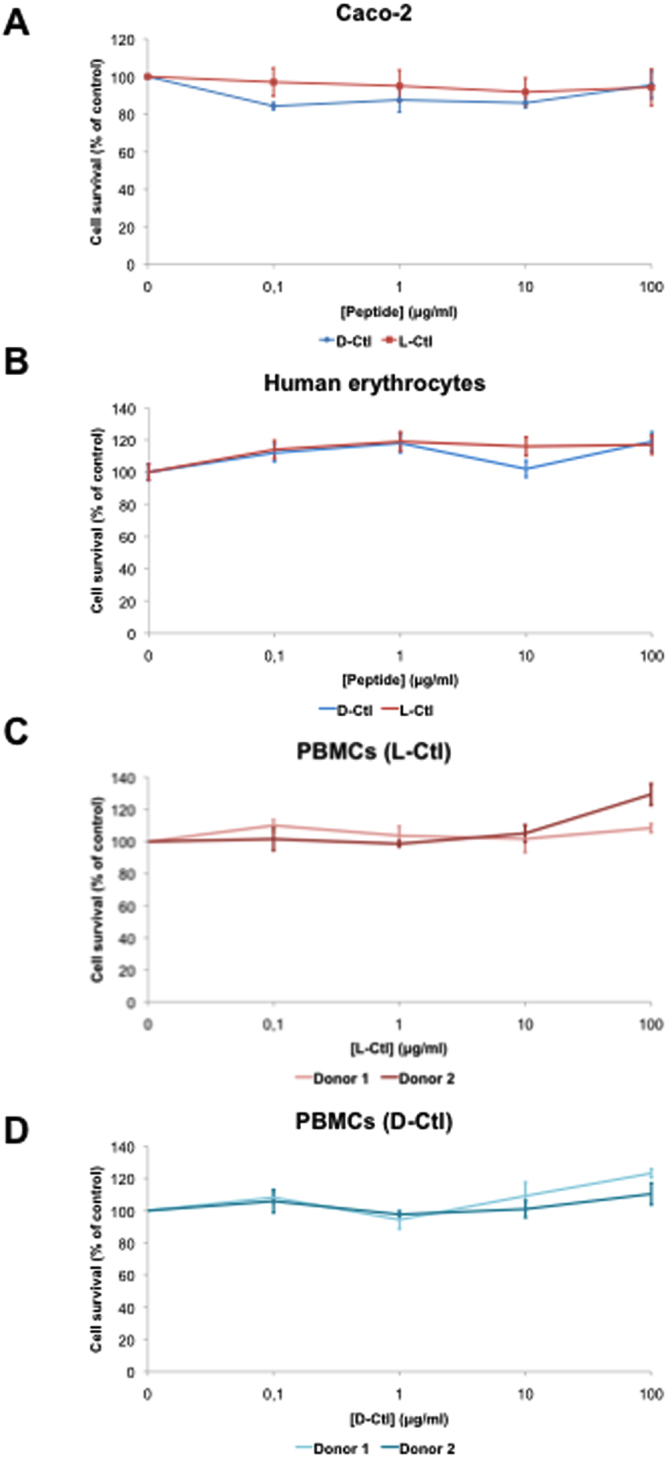
Cytotoxicity assays of D-Ctl and L-Ctl. The cytotoxicity of D-Ctl and L-Ctl on Caco-2, a human intestinal epithelial cell line (**A**) and PMBCs (**C** and **D**) was assessed at the indicated concentrations for 72 hours. Red blood cells haemolysis was evaluated after a one-hour treatment with the indicated concentrations of D-Ctl or L-Ctl (**B**). Each figure corresponds to a mean of at least two independent experiments.

One of the major side effects of conventional antimicrobials, but also several HDPs, is to alter the intestinal homeostasis by damaging the intestinal epithelial barrier^21^. To verify whether D-Ctl affects the integrity of the intestine epithelium, we assessed the cytotoxicity of D-Ctl towards Caco-2 cells, a human intestinal epithelial cell line. As shown in Fig. 2A, no cytotoxicity was measured after 72 hours of incubation with neither D-Ctl nor L-Ctl at concentrations up to 100 μg/mL.

In order to be administered as a systemic therapy, antimicrobials should not interfere with blood cells homeostasis. Subsequently, we assessed whether D-Ctl was toxic towards human erythrocytes but also human peripheral blood mononuclear cells (PBMCs). For haemolytic assays, D-Ctl or L-Ctl was incubated with human erythrocytes at concentrations ranging from 0 to 100 μg/mL. No cell lysis was observed at all, demonstrating that neither D-Ctl nor L-Ctl is haemolytic, even at concentrations higher than its MICs **(**Fig. 2B **)**. Similarly, no cytotoxicity was detected in PBMCs following an exposure of 72 hours with D-Ctl or L-Ctl at concentrations up to 100 μg/mL **(**Fig. 2C and D **)**.

**Figure 3:**
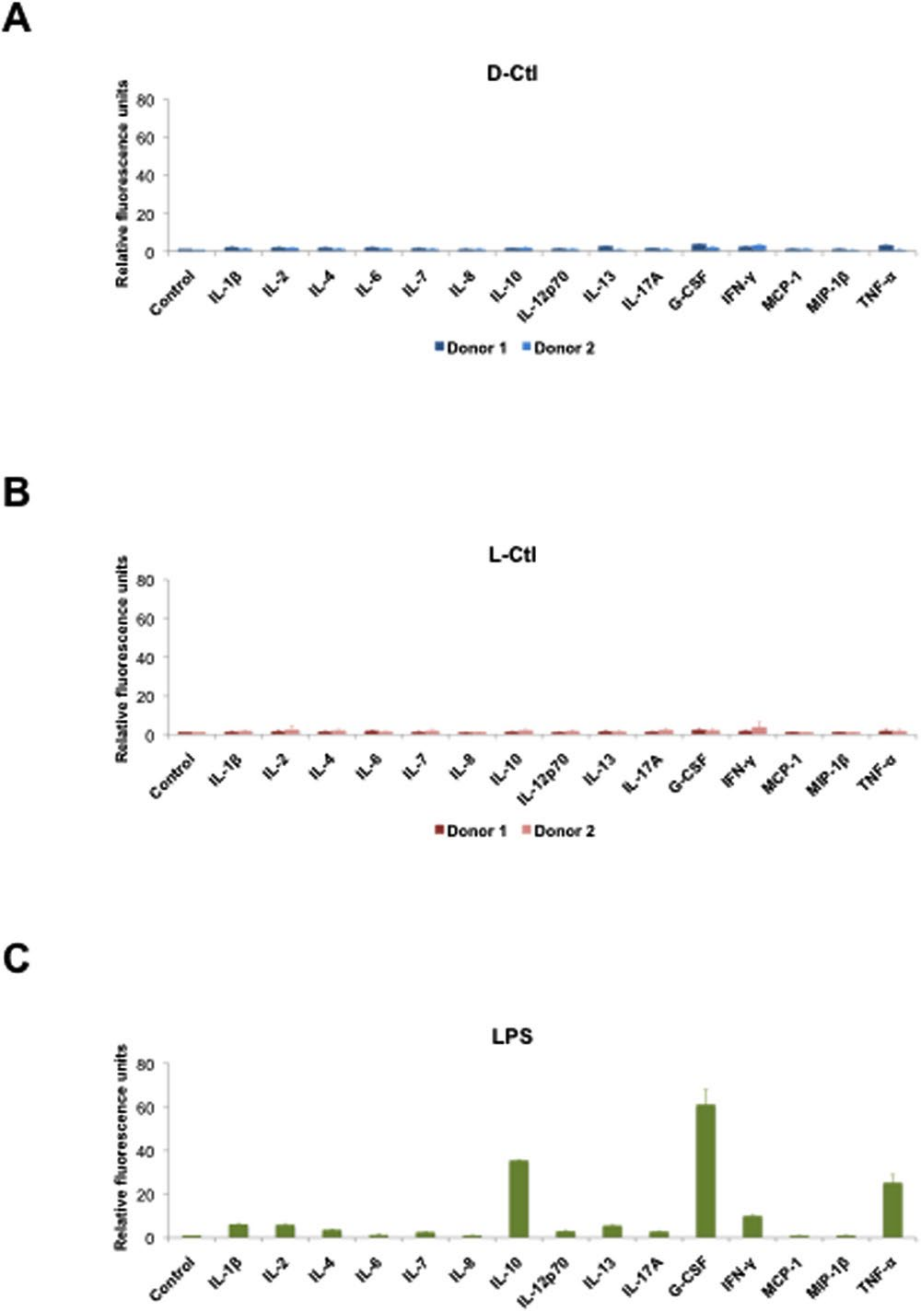
Cytokine release assay following treatment of PBMCs with D-Ctl or L-Ctl. Cells from healthy volunteers were treated with D-Ctl (**A**), L-Ctl (**B**) or LPS (**C**) for 24 hours and the indicated cytokines levels were evaluated in the cell supernatant using the Bio-Plex® technology.

In addition, an antimicrobial drug candidate should not trigger immunogenicity. To verify whether D-Ctl influences the immune system, we performed a cytokine release assay. To this aim, human PBMCs were treated with D-Ctl for 24 hours and cytokines were quantified after 24 hours in the cell supernatant using the Bio-Plex® technology (Bio-Rad). As indicated in Fig. 3A and B, no significant cytokine release was observed following D-Ctl or L-Ctl treatment. As a control, PBMCs were treated with LPS in the same conditions, resulting in the release of a broad spectrum of pro-inflammatory cytokines such as TNFα, G-CSF and IFNγ but also the anti-inflammatory cytokine IL-10 **(**Fig. 3C **)**. This result indicates that neither D-Ctl nor L-Ctl is associated with major cytokine release.

### D-Ctl is more resistant to degradation by secreted bacterial proteases than L-Ctl

**Figure 4:**
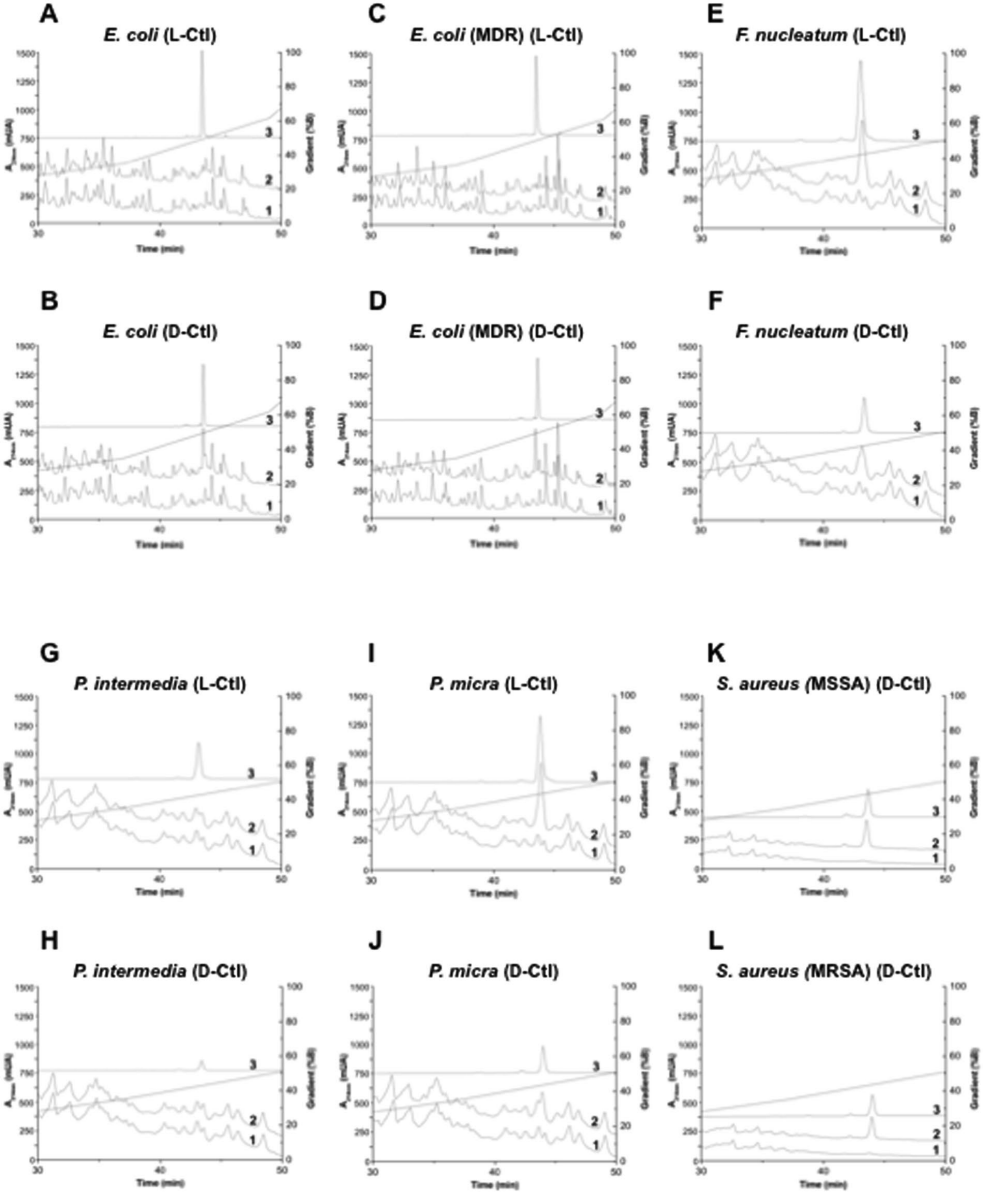
Stability of D-Ctl and L-Ctl towards proteases secreted by different bacterial strains. Supernatants from *E*. *coli* wild type (**A** and **B**), *E*. *coli* MDR (**C** and **D**), *F*. *nucleatum* (**E** and **F**), *P*. *intermedia* (**G** and **H**), *P*. *micra* (**I** and **J**), *S*. *aureus* methicillin sensitive (MSSA) (**K**), *S*. *aureus* methicillin resistant (MRSA) (**L**) were incubated with D-Ctl or L-Ctl, as indicated, for 24 hours. Peptide stability was then assessed by HPLC. Chromatograms 1 correspond to supernatant only, chromatograms 2 correspond to supernatant and peptide and chromatograms 3 corresponds to peptide only.

Linear L-peptides with α-helical structures are usually susceptible to proteolysis. As an example, V8 and aureolysin, two proteases secreted by *S*. *aureus* are responsible for the cleavage of the host defence peptide LL-37 and therefore contribute to bacterial survival^22^. The specific spatial configurations of the cleavage sites for these enzymes are not present in D-peptides although these peptides might be cleaved by non-specific hydrolysis during enzymatic digestion. Subsequently, we assessed the sensitivity of D-Ctl to secreted bacterial proteases by HPLC. To this aim, different bacterial supernatants were incubated with D-Ctl (or L-Ctl as a control) for 24 hours at 37 °C. As depicted in Fig. 4, D-Ctl was not degraded in none of the bacterial supernatants tested **(**Fig. 4B,D,F,H,J,K and L **)**. In contrast, L-Ctl was degraded in the presence of secreted proteases from *E*. *coli* wild type **(**Fig. 4A **)** and MDR **(**Fig. 4C **)** but not *F*. *nucleatum* **(**Fig. 4E **)**, *P*. *intermedia* **(**Fig. 4G **)** and *P*. *micra* **(**Fig. 4I **)**. Of interest, in a previous study, we demonstrated that L-Ctl was also stable in the supernatant of MSSA and MRSA^18^. Consequently, the change in conformation between L-Ctl and D-Ctl does not affect their sensitivity towards secreted bacterial proteases, except for *E*. *coli* wild type and MDR.

### D-Ctl dramatically damaged the cell wall of *E*. *coli* MDR

**Figure 5:**
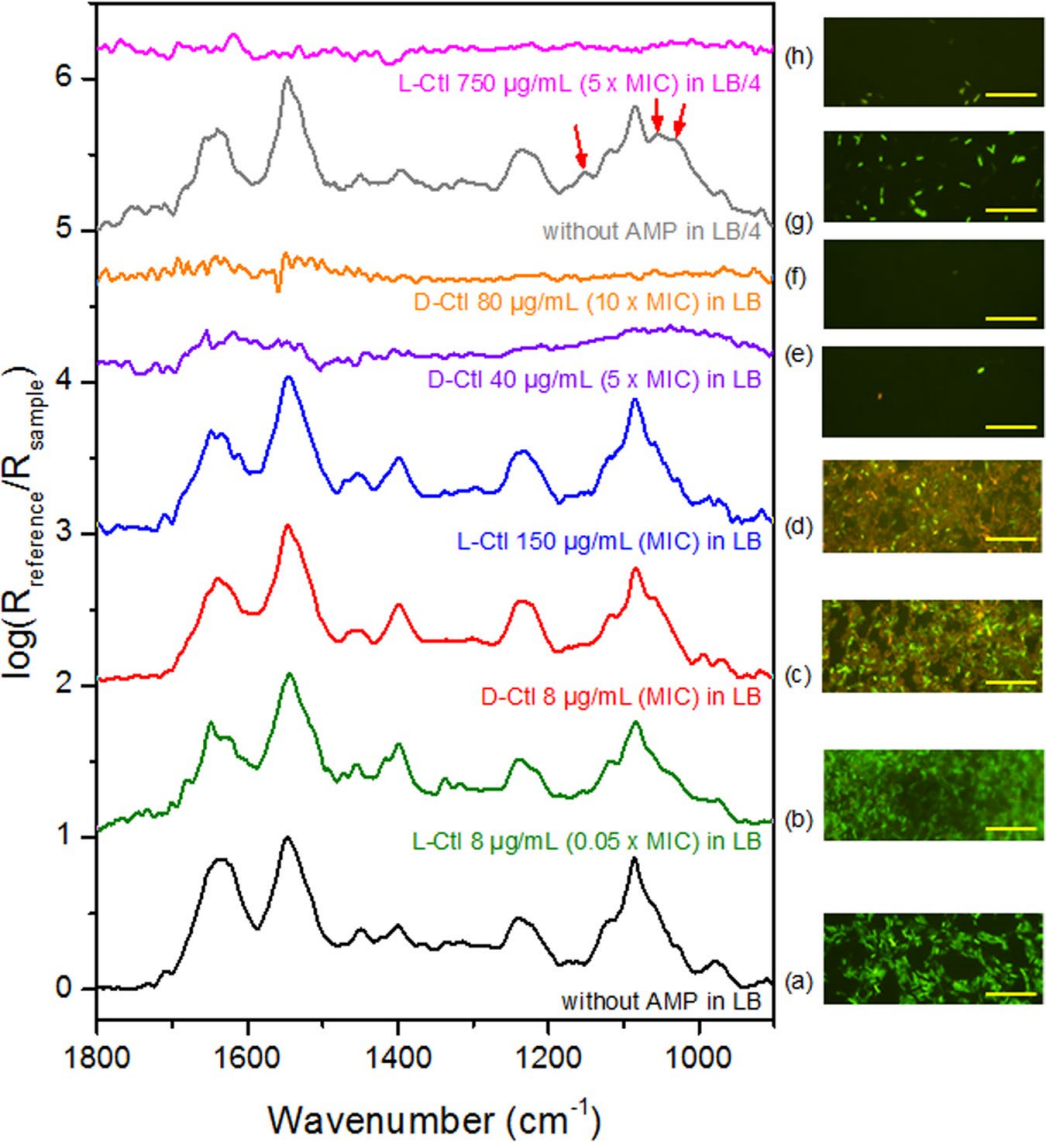
Spectral fingerprints of *E*. *coli* MDR. Left panel: IR-ATR spectra of planktonic *E*. *coli* MDR incubated with or without L and D conformers of Ctl during 20 hours. The spectra are normalized to one with respect to the Amide II band. Offsets of spectra are used for clarity. Right panel: Corresponding representative epifluorescence images of *E*. *coli MDR* after incubation with or without L and D conformers of Ctl during 20 hours. Bar: 20 µm.

**Figure 6:**
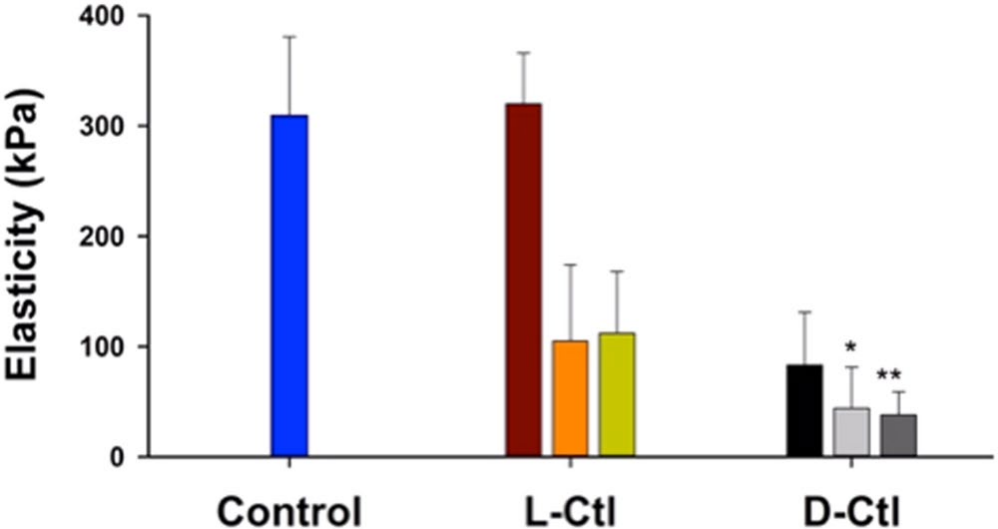
Elasticity of *E*. *coli* MDR treated with D-Ctl or L-Ctl for 20 hours. *And **refer to data obtained after only 3 hours and 0.8 hours of treatment, respectively. Bars for L-Ctl correspond to the average elasticity of bacteria subjected to antimicrobial peptide treatments performed at concentrations of 8, 150 and 750 µg/mL, respectively. For D-Ctl, the bars correspond to the average elasticity of bacteria subjected to the peptide at concentrations of 8, 40 and 150 µg/mL, respectively.

To have a first insight on the mechanism of action of both peptides, suspensions of *E*. *coli* MDR (DO_600_ = 0.1, ~6 × 10^6^ bacteria/mL) were subjected or not (as a control experiment) to the action of L-Ctl and D-Ctl during 20 hours at several initial concentrations (0.05x MIC, 1x MIC, 5x MIC, and 10x MIC). Figure 5(a and g) shows the infrared spectra of the bacteria cultivated in LB and LB/4 media without peptide. The spectral fingerprints are characteristic of live bacteria^23^. In LB/4, the additional biosynthesis of glycogen can be observed (red arrows, Fig. 5g) probably due to a lack of some nutrients with respect to carbon^24^. The corresponding epifluorescence images (next to the infrared spectra) after *Bac*Light^TM^ staining show a green fluorescence suggesting intact cell membranes. The average elasticity assessed by AFM force measurements was 310 ± 71 kPa **(**Fig. 6 **)** that was in line with previous data obtained on the same strain^25^.

At 8 µg/mL for both enantiomers, the infrared spectral features were very similar to those recorded for the untreated bacteria **(**Fig. 5a,b and c **)**, suggesting that the metabolic activity of the bacteria was not or poorly modified. However, some differences in the corresponding epifluorescence images were observed. Whereas bacteria treated by L-Ctl showed only a green fluorescence, those treated with D-Ctl at the same concentration showed some green bacteria but also a lot of orange/red bacteria. This result suggested that the membranes of the bacteria were not damaged by L-Ctl but were damaged by D-Ctl for a lot of bacteria. The mechanical properties of the bacteria reported in Fig. 6 showed that L-Ctl did not significantly impact the cell wall elasticity (320 ± 46 kPa). Consequently, the integrity of the bacterial membrane was preserved in spite of the presence of L-Ctl, in accordance with the epifluorescence results. Conversely, the treatment with D-Ctl at the same concentration dramatically reduced by a factor of 3.7 the average elasticity of the bacterial cell wall (83 ± 48 kPa). This loss of elasticity suggested that D-Ctl strongly damaged the bacterial membrane as it was already reported in the literature for other antimicrobial peptides^26–28^. These results emphasized that the action of the two enantiomers were very different at the same concentration. Whereas D-Ctl showed a very strong activity against *E*. *coli* MDR, this was not the case for L-Ctl. Indeed, for the latter the concentration was only 0.05x MIC instead of MIC for D-Ctl.

When the bacteria were treated at the MIC of L-Ctl (150 μg/mL), the infrared spectrum of the bacteria left after the treatment was very similar to the one of the non-treated bacteria **(**Fig. 5a and d **)**. This result suggested that as for D-Ctl at the MIC, the treatment with L-Ctl at the MIC did not or slightly modify the bacterial metabolism. The epifluorescence images after BacLightTM staining show a mixture of green and orange/red bacteria. It suggested that the bacterial membranes were damaged for some bacteria as it was previously observed for D-Ctl at its MIC. The calculated average elasticity was reduced by a factor of 3 with respect to the untreated bacteria (105 ± 69 kPa, see Fig. 6). The action of both enantiomers was almost the same on the membrane elasticity at their MICs.

For higher concentrations of L-Ctl and D-Ctl (at 750 µg/mL and above 40 µg/mL, respectively) no infrared spectra could be recorded **(**Fig. 5e,f and h **)**. This result was in accordance with epifluorescence images. Only very few bacteria were observed on the filters. The bacteria were almost completely lysed. In the case of L-Ctl, AFM measurements show no significant difference between the treatments performed at the MIC and at 5x MIC in terms of elasticity (112 ± 56 kPa for the latter concentration). For D-Ctl at 40 and 80 µg/mL, the bacterial elasticity could be measured only as soon as at 3 hours and 0.8 hours, respectively, because no bacteria were left after 20 hours of treatment. The average elasticity was already reduced by a factor 7 to 8 (44 ± 37 kPa and 28 ± 21 kPa, respectively, Fig. 6). Conversely to the action of L-Ctl above the MIC, the damages that occurred on the bacteria were reached earlier with D-Ctl, and they were dramatic for the cell integrity.

## Discussion

The rise of antimicrobial resistant microorganisms constitutes an increasingly serious threat to global public health. As a consequence, the efficacy of conventional antimicrobials is rapidly declining, threatening the ability of healthcare professionals to cure common infections^1,2^. Hence, the development of new antibacterial compounds with less potential to trigger resistance constitutes a public health challenge.

In the last two decades, host defence peptides have been proposed as a potential source of novel antimicrobials^12^. Although more efficient antimicrobials are currently on the market^29^, host defence peptides display numerous advantages over conventional antimicrobials, such as an incomparably broad spectrum of action, a fast mode of action and most importantly, a very low potential to induce resistance. In this study, we report the antibacterial properties of D-Ctl on a large panel of bacteria including Gram-positive and Gram-negative pathogens but also obligate and facultative anaerobes. D-Ctl is a derivative of L-Cateslytin (L-Ctl), already known for its antimicrobial properties, specifically against *S*. *aureus*. D-Ctl consists of the same sequence as L-Ctl with all levogyre (L) amino acids replaced by dextrogyre (D) amino acids. By introducing these modifications, we intended to increase the stability of the peptide towards bacterial proteases, as liability is the Achilles’ heel of peptide therapeutics. Indeed, in contrast to L-Ctl, D-Ctl cannot be degraded by cellular proteases. In accordance, our results demonstrated that D-Ctl is stable in all bacterial supernatant tested (MSSA and MRSA, *E*. *coli wild* type and MDR, *P*. *micra*, *P*. *intermedia* and *F*. *nucleatum*). Remarkably, L-Ctl was already a robust compound, resistant to the degradation by secreted proteases from *S*. *aureus* MSSA and MRSA^18^, *P*. *micra*, *P*. *intermedia* and *F*. *nucleatum* but degraded in the supernatant of *E*. *coli* wild type and MDR.

As expected, D-Ctl was much more efficient than L-Ctl with a difference in the MIC ranging from 1.7 (MSSA) to 17.9 folds (*E*. *coli* MDR). Active against both Gram-positive and Gram-negative bacteria, D-Ctl could be considered as a broad-spectrum antimicrobial. However, a larger panel of pathogens remain to be screened to validate such an assumption. Nevertheless, D-Ctl was specifically efficient on *E*. *coli* wild type and MDR with a MIC of 8.0 µg/mL and 8.4 µg/mL, respectively. Overall, the MICs of D-Ctl were comparable with the ones of LL-37 and its truncated mimetics KE-18 and KR-12 (8.4 to 19.3 µg/mL for *S*. *aureus* and 2.1 to 9.8 µg/mL for *E*. *coli*)^30^ but also of human β-defensins 2 and 3, which ranged between 1.4 µg/mL and >250 µg/mL depending on the bacterial strain^31^. When compared to the antimicrobial of reference for each pathogen, antimicrobials were still more efficient than D-Ctl except for *E*. *coli* wild type where the efficiency of D-Ctl (MIC = 8.0 µg/mL) was comparable with ampicillin (MIC = 7.0 µg/mL) and much higher than kanamycin (MIC = 21.6 µg/mL). However and of high interest, the potential for *E*. *coli* to develop resistance to D-Ctl under selective pressure was not detectable for D-Ctl, unlike ampicillin and cefotaxime (three fold MIC increase for both antimicrobial over 24 days).

Combination antibacterial therapy is frequently used to prevent or delay the emergence of resistance^32^. Interestingly, D-Ctl is not only a strong antimicrobial candidate against *E*. *coli*, but it can also be used in conjunction with conventional antimicrobials to enhance their antibacterial activity against other pathogens. As a matter of fact, here we report the synergistic effect of D-Ctl and amoxicillin against *P*. *micra* and *P*. *intermedia*. Furthermore, D-Ctl in combination with cefotaxime, methicillin or amoxicillin displayed an additive antibacterial effect against *E*. *coli* MDR, *S*. *aureus* and *F*. *nucleatum*, respectively. As a result of these associations, the concentration of conventional antimicrobials could be remarkably decreased from a factor two to four with potential implications on bacterial resistance.

Remarkably, the antibacterial activity of D-Ctl was not associated with cellular toxicity and does not interfere with the production of cytokines from LPS-stimulated PBMCs. These toxicology outcomes constitute a valuable point towards the use of D-Ctl as a new antimicrobial against *E*. *coli* infections. Indeed, the powerful antibacterial activity of most antimicrobials currently on the market is balanced by detrimental side effects. Specifically, fluoroquinolones, the antimicrobials of reference against *E*. *coli* infections are associated with immunomodulation, severe nephrotoxicity and tendinopathies^33,34^. Besides, D-Ctl was insensitive to proteases secreted by targeted pathogens. This property of D-Ctl was expected, as there is no L-amino acid within its structure.

Mechanism by which D-Ctl exerts its antibacterial activity was deciphered by physico-chemical methods. From the infrared data, it is suggested that the bacterial metabolism was not or poorly impacted. However the bacterial membrane was permeabilized as it was shown by the epifluorescence images after *Bac*Light^TM^ staining. From the drastic decrease of the cell wall elasticity, it can be also suggested that the bacterial cell wall is highly damaged, and action of D-Ctl leads to loss of cytosol until the bacterial lysis and the death of the bacteria. Here we showed that the rate of the antimicrobial action and the minimum amount of peptide molecules necessary to reach the cell lysis are strongly dependent on the conformation of the peptide. Surprisingly, our results demonstrated that the D-conformer had the most efficient action for the lowest MIC (by a factor of around 20), contrary to previous studies that did not show such a significant difference in antimicrobial activity of L- and D-conformers^35,36^.

In the last decade, there have been a few HDPs entering clinical trials, specifically cathelicidins and defensins natural peptides or derivatives such as LL-37, MBI-226 (studies NCT00211523, NCT00211497 and NCT00027248 for the prevention of central venous catheter-related bloodstream infections and acne) and PMX-30063 (study NCT01211470 for acute bacterial skin and skin-structure infection). However, the clinical and commercial development of these peptide-based drugs has some limitations such as high cost of production, susceptibility to proteases and cytotoxicity. For example, the human cathelicidin LL-37 enhances apoptosis of epithelial cells, smooth muscle cells and T cells at levels above 10 µM^37^. Besides being cytotoxic, LL-37 is also sensitive to protease cleavage, leading to the abolishment of its antimicrobial properties^38^. Defensins have also been extensively considered as an alternative to classical antimicrobials. However, the main limitation to their use as therapeutics is the lack of efficient production methods due to their complex secondary and tertiary structures^39,40^. In this context, D-Ctl presents many assets compared to other peptide-based drugs. Indeed, D-Ctl is short (15 amino acids) and linear, which makes it really easy to produce. Moreover, the use of a D-peptide emerges as a fruitful strategy to avoid degradation by secreted bacterial proteases. To put it in a nutshell, D-Ctl emerges as a potent, safe and robust antimicrobial with undetectable susceptibility to resistance, which makes it an attractive candidate for biopharmaceutical development. However, for an eventual entry into humans, a full assessment of safety pharmacology and drug toxicology will have to be conducted.

## Methods

### Peptide synthesis

The chemically synthesized peptides corresponding to L-Cateslytin (L-Ctl) and D-Cateslytin (D-Ctl) (RSMRLSFRARGYGFR, purity >95%) were purchased from Proteogenix.

### Microorganisms and mammalian cell cultivation

*Escherischia coli* (ATCC® 25922™), *Staphylococcus aureus* (ATCC® 25923™), *Fusobacterium nucleatum* (ATCC® 49256™), *Prevotella intermedia* (ATCC® 49046™) and *Parvimonas micra* (ATCC® 33270™) were purchased from ATCC. *E*. *coli* K-12 mutant multidrug resistant (MDR) was kindly provided by the Institut Pasteur of Paris. This strain was constructed from *E*. *coli* MG1655 (*E*. *coli* genetic stock center CGSC#6300). It is resistant to specific antimicrobials such as ampicillin, chloramphenicol, and kanamycin^25^. The *S*. *aureus* Methicillin Resistant (MRSA) S1 strain was kindly provided by Dr Gilles Prévost (University of Strasbourg)^18^. Microorganisms were cultured according to the manufacturer’s or the owner’s instructions in their respective media: Luria Bertani broth (Sigma) was used for *E*. *coli* strains, Mueller Hinton broth (Difco) for *S*. *aureus* strains and Anaerobe Basal broth (Oxoid) for *F*. *nucleatum*, *P*. *intermedia* and *P*. *micra*.

The Caco-2 cell line (ATCC® HTB-37™) was kindly provided by Dr Benoît Frisch (UMR 7199 CNRS University of Strasbourg) and cultured at 37 °C in a 5% CO2 humidified incubator in Eagle’s Minimum Essential Medium (Thermo Fisher Scientific) supplemented with 20% bovine calf serum and 1% penicillin/streptomycin. Human Peripheral Blood Mononuclear Cells (PBMC) from healthy volunteers were obtained from the blood transfusion centre of Strasbourg (Etablissement Français du Sang, Strasbourg) and isolated by density gradient centrifugation using Lymphoprep™ (Stemcell Technologies). PMBC were then maintained in AIM V® medium (Thermo Fisher Scientific) at 37 °C in a 5% CO2 humidified incubator.

### Minimum inhibitory concentration (MIC) determination

The MIC was determined by broth microdilution. An overnight culture of each bacterial strain was diluted (approximately to OD_600_ = 0,001) and microorganisms were plated in 96-well plates in the presence of different concentrations of antimicrobials, D-Ctl or L-Ctl alone or in combination. Three technical replicates were performed for each condition. After 24 hours of incubation, the microorganism growth was assessed by optical density OD_600_ using a Multiskan™ EX microplate spectrophotometer (Thermo Fisher Scientific). The MIC, defined as the lowest concentration of a drug alone or in combination able to inhibit 100% of the inoculum, was determined from a modified Gompertz model as described in Lambert *et al*.^41^. Each experiment was performed with at least three biological replicates.

### Haemolytic assays

The lysis of red blood cells was monitored by the release of haemoglobin to the extracellular environment. Whole blood from one healthy volunteer was obtained from the blood transfusion centre of Strasbourg (Etablissement Français du Sang, Strasbourg). Cells were then washed twice with PBS (800 g, 10 min), resuspended in 1 mL of PBS and incubated with D-Ctl or L-Ctl at different concentrations (0–100 μg/mL) for 1 hour at 37 °C. As a positive control, total lysis of red blood cells was obtained by incubating the cells with 0.1% SDS. For each condition, three technical replicates were performed. After the incubation, cells were centrifuged at 800 g for 10 min and the level of haemoglobin released in the supernatant was determined by optical density OD_420_ using a Multiskan™ EX microplate spectrophotometer (Thermo Fisher Scientific).

### Cell viability assays

The MTT [3-(4,5-dimethylthiazol-2-yl)-2,5 diphenyl tetrazolium bromide] assay was used to assess the cytotoxicity of D-Ctl and L-Ctl. Cells in their exponential phase of growth were seeded into a 96-well plate at 1 × 10^6^ cells/mL prior being treated with a tenfold serial dilution of D-Ctl or L-Ctl. Three technical replicates were performed for each condition. After 72 hours incubation, MTT (Sigma-Aldrich) was added to each well at a final concentration of 0.25 mg/mL. Cells were then incubated for an additional 2 hours at 37 °C in a 5% CO2 humidified incubator and lysed with isopropanol/HCl (96:4, v/v). Cell cytotoxicity was then assessed by optical density OD_570_ using a Multiskan™ EX microplate spectrophotometer (Thermo Fisher Scientific). Each experiment was performed with at least three biological replicates.

### Cytokine release assays

The following cytokines: G-CSF, GM-CSF, IFN-γ, IL-1β, IL-2, IL-4, IL-5, IL-6, IL-7, IL-8, IL-10, IL-12, IL-13, IL-17, MCP-1, MIP-1β, TNF-α were measured using the Bio-Plex® Multiplex Immunoassay system (Bio-Rad). In brief, human PBMCs were prepared as previously described and treated for 24 hours with D-Ctl (60 μg/mL), L-Ctl (60 μg/mL) or LPS (5 μg/mL). Three technical replicates were performed for each condition. Supernatants were then filtered and assessed for cytokine dosage according to the manufacturer’s instructions.

### Resistance acquisition assays

An *E*. *coli* (ATCC® 25922™) culture was sequentially diluted every day in the presence of the different antibacterial compounds: D-Ctl, ampicillin or cefotaxime at ½ MIC during 24 days. The changes in the MICs values were determined as previously described by broth microdilution at the indicated times. The experiment was performed with three technical replicates.

### Peptide stability assays towards secreted bacterial proteases

Bacterial supernatant was prepared as follows: a single colony of each strain was resuspended in 5 mL of culture medium as indicated above and incubated at 37 °C overnight. The culture was then centrifuged at 10000 g for 1 min and the supernatant was filtered using a 0.22 mM MillexH-GV (Millipore, Carrigtwohill, Ireland). An aliquot of each supernatant was incubated at 37 °C for 48 hours. Absence of growth was interpreted as lack of viable microorganism. 400 μL of supernatant was then incubated with or without each peptide of interest at 37 °C for 24 hours. As a control, each peptide was incubated in water at 37 °C for 24 hours. Samples were then separated using a Dionex HPLC system (Ultimate 3000; Sunnyvale, CA USA) on a Nucleosil reverse-phase 300–5C18-column (46250 mm; particle size: 5 mm; porosity, 300 Å) (Macherey Nagel, Hoerdt, France). Absorbance was monitored at 214 nm and the solvent system consisted of 0.1% (v/v) TFA in water (solvent A) and 0.09% (v/v) TFA in 70% (v/v) acetonitrile-water (solvent B). Elution was performed at a flow rate of 700 mL/min with a gradient of solvent B as indicated on the chromatograms.

### Planktonic *E*. *coli* suspensions for physicochemical analysis

The bacterial model used for the physicochemical analysis (AFM, infrared spectroscopy and epifluorescence microscopy) is *E*. *coli* MDR. Bacteria were cultured in Luria Broth (Miller, Fluka) at 25 g/L (LB) or at 6.25 g/L (LB/4) in deionized water (Purelab Option, ELGA). All the cultures were incubated in a water bath shaker (Inova 3100, New Brunswick Scientific) at 37 ± 1 °C and under continuous agitation at 160 rpm. After an overnight subculture (16 hours, with ampicillin and kanamycin), bacteria were cultured in 200 mL of LB medium (without antimicrobials) with an initial optical density at 600 nm (OD_600_, measured with a cell density meter Biochrom AG, Fisherbrand) of 0.050 ± 0.005.

For epifluorescence and infrared spectroscopy analyses, the antimicrobial assays against planktonic *E*. *coli* MDR were performed in duplicate in sterile 96-well plates (Nunc) in a final volume of 200 mL. When the optical density of the bacterial culture reached an OD_600_ value between 0.5 and 0.6 (bacteria were at the end of the exponential phase), the suspension was diluted in LB or LB/4 to give an OD_600_ = 0.10 ± 0.01. The necessary volume of the stock solution of the peptide at 1 g/L was spotted in the bacterial suspension. Sterility and growth controls were sterile LB and LB/4, and a bacterial suspension without peptide, respectively. The plate was incubated for 20 hours at 22 °C.

### Epifluorescence optical microscopy

Planktonic bacteria were analysed by fluorescence microscopy using the *Bac*Light^TM^ stain kit (L7012, Molecular Probes, Eugene, USA) in order to determine the permeability of the cells in the absence and presence of the peptide. This kit contains two nucleic acids dyes: SYTO 9 (excitation/emission maxima: 480/500 nm) that penetrates all the cells, and propidium iodide that penetrates only cells with damaged membranes (excitation/emission maxima: 490/635 nm). Therefore, bacteria with intact membranes fluoresce green, while bacteria with damaged membranes fluoresce red. After 20 hours of incubation, 200 µL of the 24 hours-old bacterial suspension were mixed with 300 µL of *Bac*Light^TM^ solution (15 µL of the reconstructed *Bac*Light^TM^ solution as described by the manufacturer in 300 µL of sterile water), and stained for 20 min in the dark at 22 ± 1 °C. The suspension was then filtrated with 0.2 µm black filters (Millipore, GTBP04700) and rinsed three times with sterile water to eliminate excess *Bac*Light^TM^. The sample was mounted in *Bac*Light^TM^ mounting oil as described by the manufacturer. Both fluorescences were viewed simultaneously with the 100x oil immersion objective of an Olympus BX51 microscope equipped with an Olympus XC50 camera.

### ATR-FTIR spectroscopy

ATR-FTIR spectra were recorded between 4000 and 800 cm^−1^ on a Bruker Vertex 70 v spectrometer equipped with a KBr beam splitter and a DTGS detector, and driven by the OPUS 7.5 software. The resolution of the single beam spectra was 4 cm^−1^. A nine-reflection diamond ATR accessory (Durasampl*IR*™, SensIR Technologies, incidence angle: 45°) was used for acquiring spectra. The number of bidirectional double-sided interferogram scans was 200, which corresponds to a 2 min accumulation. All interferograms were Fourier processed using the Mertz phase correction mode and a Blackman-Harris three-term apodization function. No ATR correction was performed. Measurements were performed at 21 ± 1 °C in an air-conditioned room. 50 µL of the bacterial suspensions in their culture media was put on the ATR crystal. Half of the suspension was centrifuged at 8000 rpm during 5 min and the supernatant was used to remove the spectral background. Water vapour subtraction was performed when necessary.

### AFM mechanical properties measurements

AFM experiments were carried out using a MFP3D-BIO instrument (Asylum Research Technology, Oxford Instruments Company, Mannheim, Germany). Silicon nitride cantilevers of conical shape were purchased from Asylum Research Technology (Olympus TR400 PSA, Mannheim, Germany). The spring constants of the cantilevers measured using the thermal noise method were found to be 0.02–0.03 nN/nm. Experiments were performed in triplicate in PBS at room temperature. The nanoindentation method was used to determine the Young’s modulus from the force vs. indentation curves. Mechanical properties were obtained by recording a grid map of 50-by-50 force curves on several bacterial clusters containing at least 10 bacteria electrostatically immobilized onto PEI coated glass substrate. The maximal loading force was 4 nN, the piezodrive was fixed to 2 µm and the approach rate was 2 µm/s. The histograms corresponding to the statistic distribution of the Young modulus were estimated from the analysis of the approach curves according to the Sneddon model^42,43^ where δ is the indentation depth, ν the Poisson coefficient, *R* is the curvature radius of AFM-tip apex and *f*_*BECC*_ the bottom effect correction described by Gavara et Chadwick^42^. All the FVI were analysed by mean of an automatic Matlab algorithm described elsewhere^44^. Bacteria were then exposed to various L-Ctl concentrations (8, 150 and 750 µg/mL) and also to various D-Ctl concentrations (8, 40 and 80 µg/mL) in PBS buffer at 22 °C for 20 hours. Mechanical properties were measured by AFM in force mapping mode at indentation rate of 2 µm/s and the average values correspond to at least 500 force curves taken from at least 10 bacteria. For bars labelled with * and ** the corresponding values were obtained after only 3 and 0.8 hours of peptide exposure, respectively. Of notice, beyond these exposure periods all bacteria were too damaged and not enough for relevant measurements.

## Electronic supplementary material

Supplementary Dataset 1
