# Supplementary material for: D-Cateslytin, a new antimicrobial peptide with therapeutic potential
Source: Sci Rep. 2017 Nov 9;7:15199. doi: 10.1038/s41598-017-15436-z (PMC5680178; doi:10.1038/s41598-017-15436-z)

### D-Cateslytin, a new antimicrobial peptide with therapeutic potential

Abdurraouf Zaet^1,2^, Pauline Dartevelle^1,2^, Fadoua Daouad^1,2^, Claire Ehlinger^1,2^, Fabienne Quilès^3,4^, Grégory Francius^3,4^, Christian Boehler^1,2^, Camille Bergthold^1,2^, Benoît Frisch^5^, Gilles Prévost^6^, Philippe Lavalle^2^, Francis Schneider^1,2,7^, Youssef Haïkel^1,2^, Marie-Hélène Metz-Boutigue^2^, Céline Marban^1,2*^

^1^ Université de Strasbourg, Faculté de Chirurgie Dentaire, 3 rue Sainte Elisabeth 67000, Strasbourg, France

^2^ Inserm UMR 1121, 11 rue Humann, 67085 Strasbourg, France, Fédération de Médecine Translationnelle de Strasbourg

^3^ Université de Lorraine, Laboratoire de Chimie Physique et Microbiologie pour l'Environnement, LCPME, UMR 7564, 54600 Villers-lès-Nancy, F-54600, France

^4^ CNRS, Laboratoire de Chimie Physique et Microbiologie pour l’Environnement, LCPME, UMR 7564, 54600 Villers-lès-Nancy, F-54600, France

^5^ Laboratoire de Conception et Applications des Molécules Bioactives, Faculté de Pharmacie, UMR 7199 CNRS/Université de Strasbourg, 74 Route du Rhin, 67401 Illkirch, France

^6^ Université de Strasbourg, CHRU Strasbourg, Fédération de Médecine Translationnelle de Strasbourg, VBP EA/7290, 67000 Strasbourg, France

^7^ Service de Réanimation Médicale, Hôpital de Hautepierre, Hôpitaux Universitaires de Strasbourg, France

* Corresponding author: celinemarban@gmail.com

**SUPPLEMENTAL FIGURE LEGENDS**

**Supplementary Figure S1: Antibacterial activity of D-Ctl and L-Ctl against pathogenic microorganisms.** The percentage of growth inhibition of *E. coli* wild type, *E. coli* MDR, *F. nucleatum*, *P. intermedia*, *P. micra*, *S. aureus* methicillin sensitive (MSSA), *S. aureus* methicillin resistant (MRSA) in the presence of different concentrations of D-Ctl (right panel) or L-Ctl (left panel) was determined by broth microdilution assays. Then, the MIC was determined using a modified Gompertz function. Experiments were performed with biological replicates.

**Supplementary Figure S2: Antibacterial activity of conventional antimicrobials against pathogenic microorganisms.** The percentage of growth inhibition of *E. coli* wild type, *E. coli* MDR, *F. nucleatum*, *P. intermedia*, *P. micra*, *S. aureus* methicillin sensitive (MSSA), *S. aureus* methicillin resistant (MRSA) in the presence of different concentrations of antimicrobials was determined by broth microdilution assays. The MIC was calculated using a modified Gompertz function. Experiments were performed with biological replicates.

**Supplementary Figure S3: Antibacterial activity of D-Ctl in combination with conventional antimicrobials against pathogenic microorganisms.** The percentage of growth inhibition of the indicated pathogens in the presence of different concentrations of antimicrobials was determined by broth microdilution assays. The MICs, defined as the lowest concentrations of each drug or the combo able to inhibit 100% of the inoculum, were used to calculate the FIC index of each combination. Each experiment was performed at least in duplicate. (CFT=cefotaxime, AMX=amoxicillin, MET=methicillin, VCN=vancomycin).


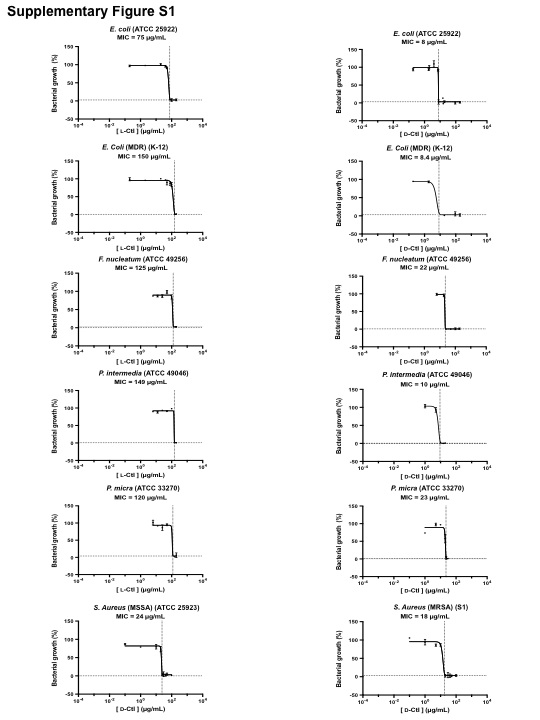


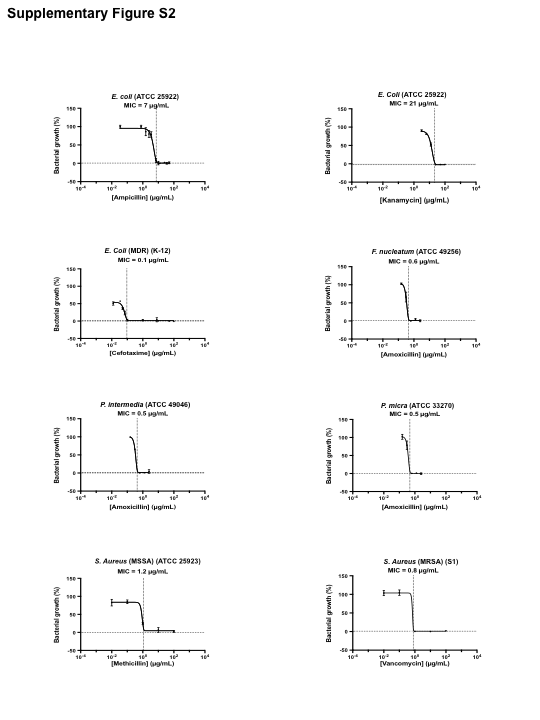


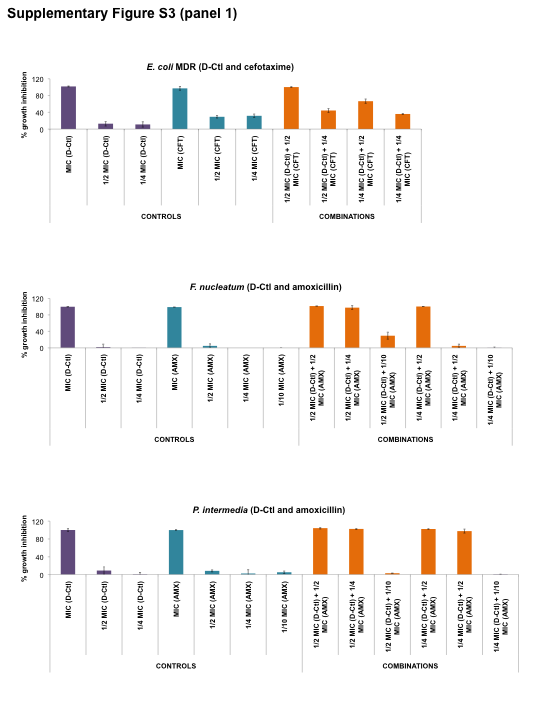


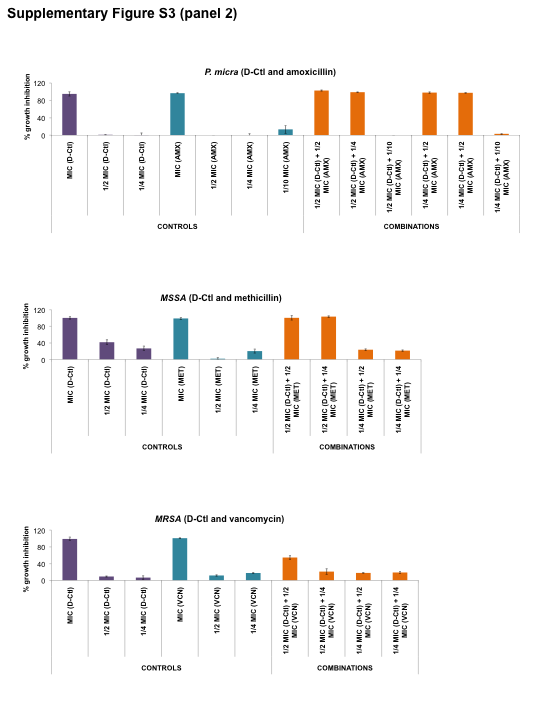

Supplement: Supplementary file 1 — Supplementary Dataset 1 [file 41598_2017_15436_MOESM1_ESM.doc]
